# Supplementary material for: Carbon isotope discrimination and the factors affecting it in a summer maize field under different tillage systems
Source: PeerJ. 2022 Feb 11;10:e12891. doi: 10.7717/peerj.12891 (PMC8842653; doi:10.7717/peerj.12891)
Supplement: Supplemental Information 2 — The specific operating procedures for the tillage treatments [file peerj-10-12891-s002.docx]

The specific operating procedures for the tillage treatments were as follows:

Conventional tillage: maize mechanical harvesting→straw return/straw removal→base fertilizer application→disc harrow stubble removal→plowing (tillage depth 20 cm)→ridge building→wheat no-tillage planter direct sowing→wheat mechanical harvesting→straw return/straw removal→maize no-tillage iron stubble sowing.

Subsoiling: maize mechanical harvesting→straw return/straw removal→base fertilizer application→subsoiling shovel subsoiling (tillage depth 35 cm)→wheat no-tillage seeder direct sowing→wheat mechanical harvesting→straw return/straw removal→maize no-tillage stubble sowing.

Rotary tillage: maize mechanical harvesting→straw return/straw removal→base fertilizer application→rotary tillage with rotary tiller (tillage depth 10 cm)→wheat no-tillage planter direct sowing→wheat mechanical harvesting→maize no-tillage iron stubble sowing.

No-tillage: maize mechanical harvesting→straw return/straw removal→base fertilizer application→wheat no-tillage planter direct sowing→wheat mechanical harvesting→straw return/straw removal→maize no-tillage iron stubble sowing.
